# Overexpression of wheat gene *TaMOR* improves root system architecture and grain yield in *Oryza sativa*

**DOI:** 10.1093/jxb/erw193

**Published:** 2016-05-26

**Authors:** Bo Li, Dan Liu, Qiaoru Li, Xinguo Mao, Ang Li, Jingyi Wang, Xiaoping Chang, Ruilian Jing

**Affiliations:** National Key Facility for Crop Gene Resources and Genetic Improvement/Institute of Crop Science, Chinese Academy of Agricultural Sciences, Beijing 100081, China

**Keywords:** AS2/LOB protein, grain yield, root architecture, root initiation, *TaMOR*, *TaMRRP.*

## Abstract

Improved root architecture is an effective strategy to increase crop yield. We demonstrate that overexpression of transcription factor gene *MORE ROOT* (*TaMOR*) from wheat (*Triticum aestivum* L.) results in more roots and higher grain yield in rice (*Oryza sativa*). *TaMOR*, encoding a plant-specific transcription factor belonging to the ASYMMETRIC LEAVES2/LATERAL ORGAN BOUNDARIES (AS2/LOB) protein family, is highly conserved in wheat and its wild relatives. In this study, tissue expression patterns indicated that *TaMOR* mainly localizes to root initiation sites. The consistent gene expression pattern suggests that *TaMOR* is involved in root initiation. Exogenous auxin treatment induced *TaMOR* expression without *de novo* protein biosynthesis. Both *in vivo* and *in vitro* experiments demonstrated that TaMOR interacts with TaMOR-related protein TaMRRP, which contains a four-tandem-pentatricopeptide repeat motif. Overexpression of *TaMOR* led to more lateral roots in *Arabidopsis thaliana*, and *TaMOR*-overexpressing rice plants had more crown roots, a longer main panicle, a higher number of primary branches on the main panicle, a higher grain number per plant, and higher yield per plant than the plants of wild type. In general, *TaMOR-D*-overexpressing lines had larger root systems in Arabidopsis and rice, and produce a higher grain yield per plant. *TaMOR* therefore offers an opportunity to improve root architecture and increase yield in crop plants.

## Highlight

Wheat gene *TaMOR* is highly conserved in evolution and in wheat improvement, and its overexpression improves root system architecture and grain yield in rice.

## Introduction

The root system is a vital plant organ owing to its involvement in water absorption, nutrient acquisition, anchorage, propagation, storage functions, secondary metabolite synthesis, and accumulation (Saini *et al.*, 2013). Accumulated evidence indicates that improved root architecture is an efficient strategy for drought avoidance (de Dorlodot *et al.*, 2007). Higher expression of *DEEPER ROOTING 1* (*DRO1*) increases root angle and facilitates root growth in a more downward direction in rice (*Oryza sativa*). Introducing *DRO1* into a shallow-rooting rice cultivar leads to a deeper root system, enhanced drought tolerance, and stable grain yield (Uga *et al.*, 2013). Statistical data show that increased biomass and increased yield in maize (*Zea mays*) in the American Corn Belt can be attributed to improved root architecture (Hammer *et al.*, 2009). To keep pace with the increasing population and world food supply requirements under the predicted conditions of climate change, improved root systems in staple crop plants will be essential.

Root growth and differentiation are inextricably linked to plant hormones (Overvoorde *et al.*, 2010; Vanneste and Friml, 2009). Auxin, as one class of important phytohormones, attracts extensive attention because of its involvement in almost every aspect of plant growth and development, including embryogenesis, organogenesis, tissue patterning, and tropisms (Quint and Gray, 2006). Indole-3-acetic acid (IAA) is an important natural auxin, responsible for root architecture and plant development at various stages (Benkova and Bielach, 2010; Overvoorde *et al.*, 2010; Lewis *et al.*, 2011; Lavenus *et al.*, 2013). Auxin response factors (ARFs) are transcription factors (TFs) that specifically bind to TGTCTC-containing auxin response elements (AuxREs) located in the promoters of primary/early auxin response genes and mediate responses to auxin (Guilfoyle and Hagen, 2007). *ARF* genes belonging to a large gene family involved in hormone signalling are highly specific to selected cell and tissue types or developmental programmes in Arabidopsis and rice (Tian *et al.*, 2004; Wilmoth *et al.*, 2005; Wang *et al.*, 2007; Rademacher *et al.*, 2011; Qi *et al.*, 2012).

Plant-specific *ASYMMETRIC LEAVES2/LATERAL ORGAN BOUNDARIES* (*AS2/LOB*) genes, also called *LATERAL ORGAN BOUNDARIES DOMAIN* genes (*LBD* gene), were identified as putative targets of ARF in Arabidopsis, rice, and maize (Inukai *et al.*, 2005; Okushima *et al.*, 2007; Taramino *et al.*, 2007). AS2/LOB proteins contain a highly conserved LBD domain that is composed of a C-domain; a four-Cys motif, presumably required for DNA-binding; a Gly-Ala-Ser block (GAS); and a leucine-zipper-like coiled-coil motif (LX_6_LX_3_LX_6_L) as a probable protein dimerization domain (Iwakawa *et al.*, 2002; Shuai *et al.*, 2002; Majer and Hochholdinger, 2011). Previous research suggests that *AS2/LOB* genes play crucial roles in root initiation and development. *In vitro*, ARF7 binds to AuxREs in the promoters of two *AS2/LOB* genes in Arabidopsis, and ARF7 and ARF19 play a positive role in regulating lateral root formation via direct activation of *LBD/ASLs* (Okushima *et al.*, 2007; Feng *et al.*, 2012b). In rice, *CROWN ROOTLESS 1* (*CRL1*), as a member of the *AS2/LOB* gene family, encodes a positive regulator of crown and lateral root formation, and localizes in tissues where crown and lateral roots initiate (Inukai *et al.*, 2005). In maize, auxin-responsive genes *rootless concerning crown and seminal roots* (*RTCS*) and *RTCS-like* (*RTCL*), which encode LOB domain proteins, are involved in regulating the initiation and maintenance of seminal and shoot-borne root primordial formation (Taramino *et al.*, 2007; Xu *et al.*, 2015).

However, the structure and function of *AS2/LOB* genes in wheat have not been investigated. Here, we identify a *MORE ROOT* (*TaMOR*) gene series in wheat containing a typical AS2/LOB domain. *TaMOR* are highly conserved in both nucleotide and amino acid sequences. They are involved in auxin signalling in the initiation of both lateral and crown roots. Overexpression of *TaMOR* leads to more lateral roots in Arabidopsis, and *TaMOR*-overexpressing rice plants have larger root systems and increased grain yields. Furthermore, TaMOR can interact with TaMOR-related protein (TaMRRP) on the cell membrane. Our results show that *TaMOR* is a promising candidate gene for root improvement and grain yield enhancement in crops.

## Materials and methods

### Plant materials and growth conditions

Drought-tolerant common wheat (*Triticum aestivum* L.) cultivar Hanxuan 10 was used to clone genes *TaMOR-A*, *TaMOR-B*, *TaMOR-D*, and *TaMRRP*, and to detect the expression patterns of *TaMORs* in wheat. Nine diploid progenitor accessions (three A genome *T. urartu* accessions, three S (B) genome *Aegilops speltoides* accessions, and three D genome *Ae. tauschii* accessions), three tetraploid *T. dicoccoides* accessions, and 34 diverse, modern, common wheat cultivars (Supplementary Table S1) were used for genome-specific primer design and polymorphism analysis.

Rice cultivar Kitaake was used for genetic transformation. The transgenic rice and wild type (WT) were planted at the Experimental Station (39°48′ N, 116°28′ E) of the Institute of Crop Science, Chinese Academy of Agricultural Sciences, Beijing, in an area dedicated to transgenic plants. Three T_3_ homozygous transgenic rice lines carrying *TaMOR-D* were used for phenotypic assays. The rice was planted outside in peat soil that was purchased from the Institute of Vegetables and Flowers, Chinese Academy of Agricultural Sciences, and normal field management was undertaken during the growth period. Three-leaf seedlings were transplanted into plastic containers (length × width × height = 80×35×30cm) in the middle of June 2015. Each container contained two rows spaced 15cm apart, with six plants in each row. All containers were managed in randomized complete blocks with three replicates for seedling stage and maturity stage, respectively. Each replicate had three containers. The root and agronomic traits were evaluated at the stage of 3-week transplanted seedling and at maturity.

For transgenic analysis, *Arabidopsis thaliana* (ecotype Columbia) was grown in a controlled environment chamber at 22 °C, with a photoperiod of 12h/12h light/dark, a light intensity of 120 mmol m^−2^ s^−1^, and 70% relative humidity. Three T_3_ homozygous transgenic lines with relatively higher expression levels of *TaMOR-D* were used for phenotypic assays. Transgenic plants with empty vectors and the WT were used as controls. Arabidopsis seeds were sown on Murashige and Skoog (MS) medium solidified with 0.8% agar, and then vernalized for 36h at 4 °C before culturing in a controlled growth chamber. To examine root morphology, 8-day-old seedlings vertically cultured on MS medium were used for observation.

### Cloning *TaMORs*

To obtain the coding and flanking region sequences of *TaMOR*, the cDNA sequence of rice *OsCRL1* (GenBank accession: NM_-_001186339) was used as an initial template for a BLAST search against the draft genome databases of the wheat D-genome progenitor *Ae. tauschii* and A-genome progenitor *T. urartu* (Jia *et al.*, 2013; Ling *et al.*, 2013). Five pairs of primers were designed based on BLAST hits (Supplementary Table S2). Full-length genomic DNA and cDNA were obtained using the primer P-TaMOR. The PCR products were inserted into pEASY-T1 simple cloning vectors (TransGene Biotech) and were transformed into *Trans*1-T1 Phage Resistant Chemically Competent Cell (TransGene Biotech). More than 24 positive clones were sequenced. The sequences were classified into three types, designated *TaMOR-A*, *TaMOR-B*, and *TaMOR-D*, based on the sequence alignment in draft genome databases. Based on the nucleotide polymorphism in the genome sequences between *TaMOR-A*, *TaMOR-B*, and *TaMOR-D*, four genome-specific primers P-TaMOR-A1, P-TaMOR-A2, P-TaMOR-B, and P-TaMOR-D were designed to amplify *TaMOR* from the A, B, and D genome sequences, including the 5′ and 3′ flanking regions (Supplementary Fig. S1). The cDNA and genomic DNA of common wheat cultivar Hanxuan 10 were used as templates. The gene structure of *TaMOR* was determined using DNASTAR Lasergene 7.1.0 (DNASTAR, Inc., Madison, WI, USA) through alignment of the modified cDNA and genomic DNA sequences.

Screening of the wheat yeast expression library led to the target gene being amplified and sequenced. The primer P-TaMRRP (Supplementary Table S2) was designed from the sequence results to amplify the cDNA of *TaMRRP* in wheat.

### Sequence analysis

*TaMOR-A*, *TaMOR-B*, and *TaMOR-D* were cloned from the common wheat cultivar Hanxuan 10. Sequence alignment and similarity analyses were conducted by multiple sequence alignment programs available in DNASTAR. Searches of amino acid sequences of LOB proteins were performed using BLAST. By comparing sequences aligned with the Clustal W algorithm within MEGA 4.1, a neighbour-joining phylogenetic tree was constructed based on 1000 bootstrap replicates.

*TaMOR-A*, *TaMOR-B*, and *TaMOR-D* were separately cloned in nine diploid progenitor accessions and three tetraploid accessions using the genome-specific primers. The coding sequences of *TaMORs* in common wheat and progenitors were used for nucleotide polymorphism analysis and evolutionary analysis. Nucleotide diversity (π) was analysed by DnaSP 5.10 software.

*TaMOR-A*, *TaMOR-B*, and *TaMOR-D* were cloned separately using the genome-specific primers in 34 diverse, modern, common wheat cultivars. The sequences containing coding and partial flanking regions were used to analyse the conservative property during wheat improvement.

### Subcellular localization

TaMOR-D and TaMRRP were each fused upstream of GFP in the pCAMBIA1300 vector under control of the CaMV 35S promoter. The primers used for *TaMOR-D* and *TaMRRP* sub-cloning are listed in Supplementary Table S2. For observation of subcellular localization in wheat protoplasts, the constructs were transformed into wheat mesophyll protoplasts using the PEG-mediated method (Yoo *et al.*, 2007). After incubation at 25 °C for 16h, florescence signals were detected using a laser scanning confocal microscope (Leica TCS-NT, Germany). For observation of subcellular localization in tobacco (*Nicotiana benthamiana*) leaf cells, vectors with the coding regions of *TaMOR-D* and *TaMRRP*, and *GFP* as a control, were transferred into tobacco leaves through *Agrobacterium tumefaciens* (EHA105)-mediated transformation (Liu *et al.*, 2010), and detected 4 days after incubation at 22 °C in a photoperiod of 16h/8h light/dark.

### Transcriptional activity assay

**Fig. 1.**
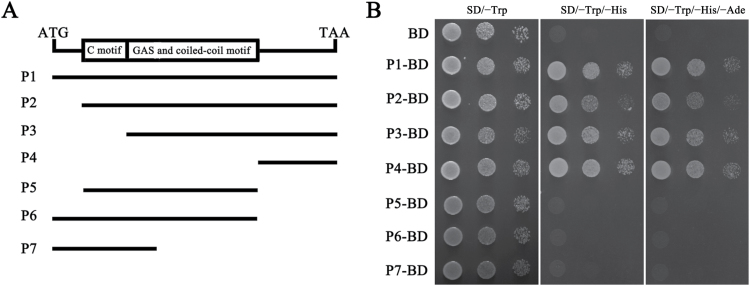
The C-terminal fragment of TaMOR-D is essential for transcriptional activation activity. (**A**) Illustration of the full-length ORF and truncations of *TaMOR-D*. According to the amino acid position of the conserved domain, the full length (P1) and six truncations (P2–P7) were subcloned into the pGBKT7 vector. (**B**) Transcriptional activation activity of TaMOR-D in a modified yeast two-hybrid assay. BD represents GAL4-binding domain (BD). P1-BD, P2-BD, P3-BD, P4-BD, P5-BD, P6-BD, and P7-BD indicate the full-length ORF and six truncations (P2–P7) introduced into the BD vector (pGBKT7). BD is the negative control.

The *Saccharomyces cerevisiae* strain AH109 and GAL4-based Matchmaker Two-Hybrid System (Clontech) were used in transcriptional activity assays. The full-length ORF of *TaMOR-D* and six truncations (shown in Fig. 1A) were cloned into pGBKT7 to produce in-frame fusions to GAL4-binding domain. The constructs were then transformed into yeast strain AH109 and cultured until optical density at 600nm = 1.0. The yeast suspension was inoculated onto SD/−Trp, SD/−Trp/−His, and SD/−Trp/−His/−Ade medium. The pGBKT7 vector was used as the negative control.

### Expression pattern analysis

Total RNA was extracted with TRizol, and cDNA was synthesized with a SuperScript® Double-Stranded cDNA Synthesis Kit (Invitrogen). Real-time quantitative PCR (qRT-PCR) was performed in triplicate with an ABI PRISM 7900 system (Applied Biosystems, USA) using the SYBR Green PCR Master Mix Kit (Takara, Japan). The primers P-TaMOR-RT and P-Tubulin (Supplementary Table S2) were used to amplify *TaMORs* and *Tubulin*.

To probe the expression pattern of *TaMOR* at various developmental stages in wheat (germination, seedling, and heading stages), different tissues including roots, root bases, leaf sheaths, leaf blades, internodes, nodes, ears, and plumules were collected. To understand the effect of exogenous auxin treatment on *TaMOR* expression in wheat, the root bases of 5-day-old wheat seedlings were collected after 0, 1, 3, 6, 12, and 24h of treatment in 1 μM IAA (Sigma Co., Germany). For inhibition of protein synthesis, 5-day-old wheat seedlings were first cultured in a solution of 50 μM cycloheximide (CHX; Sigma Co.) for 24h as a pre-treatment, and then incubated in 50 μM CHX solution, 1 μM IAA solution, 50 μM CHX and 1 μM IAA mixed solution, or H_2_O (control) for 3h. The root bases were sampled from seedlings over 13 days to examine the expression pattern of *TaMOR* following germination.

### Transgenic plants in Arabidopsis and rice

*TaMOR-D* cDNA containing the entire ORF was inserted into pCHF3 and pCUbi1390 vectors using primers P-TaMOR-D-At and P-TaMOR-D-Os (Supplementary Table S2). The two constructs were transformed into Arabidopsis and rice, respectively. T_3_ generation transgenic Arabidopsis and rice plants overexpressing *TaMOR-D* were screened with the relevant antibiotics, and reconfirmed by PCR.

### Trait evaluation of transgenic rice and Arabidopsis

Thirty plants of each transgenic rice line and WT were sampled at 3-week-seedling stage and maturity stage. Several biological and agronomic traits were measured to characterize the phenotypes of the transgenic rice, including crown root number, dry root weight, dry shoot weight, tiller number, plant height, length of main panicle, stalk diameter, primary branch number on the main panicle, 1000-kernel weight, grain number per plant, and yield per plant.

The 8-day-old Arabidopsis seedlings vertically grown on normal MS solid medium were used for phenotype observation. The number of lateral root was captured with an Epson Expression 10000XL (Epson, Japan) and counted with winRHIZO software.

### Yeast two-hybrid assay

The coding sequence of *TaMOR-D* was amplified with PCR primer P-TaMOR-D-BD (Supplementary Table S2) and the resulting products were cloned into the pGBKT7 vector. The full-length coding sequences of *TaMRRP* were amplified with primer P-TaMRRP-AD (Supplementary Table S2) and cloned into the pGADT7 vector. Yeast two-hybrid assays were based on the Matchmaker GAL4 Two-Hybrid System (Clontech). The constructs were co-transformed into yeast strain AH109. The presence of target transgenes was confirmed by growth on SD/−Leu/−Trp plates. To assess protein interactions, the transformed yeast was tested on SD/−Ade/−His/−Trp/−Leu/X-α-Gal (4mg/mL) medium. Plants were incubated at 28 °C and observed at 3 days. The experiments were triplicated.

### Bimolecular fluorescence complementation

Bimolecular fluorescence complementation (BiFC) assays of interaction between TaMOR-D and TaMRRP were performed using the *Agrobacterium*-mediated tobacco leaf transformation system. The ORFs of *TaMOR-D* and *TaMRRP* were amplified with primer pairs P-TaMOR-D-cYFP and P-TaMRRP-nYFP (Supplementary Table S2) and cloned into vectors containing the C- and N-terminals of YFP. TaMOR-D-cYFP and TaMRRP-nYFP were co-expressed in tobacco leaves. TaMOR-D-cYFP/nYFP and cYFP/TaMRRP-nYFP were co-expressed as negative controls. Florescence was detected after incubation at 22 °C for 4 days in a photoperiod of 16h/8h light/dark.

## Results

### Cloning and sequence analysis

Three copies of cDNA sequences of *TaMOR* obtained from wheat cultivar Hanxuan 10 were designated *TaMOR-A* (GenBank accession: KU158416), *TaMOR-B* (GenBank accession: KU158415), and *TaMOR-D* (GenBank accession: KU158414) according to their genomic origins. The three *TaMOR* members have the same gene structure, each containing a single exon. *TaMOR-A* encodes a protein containing 243 amino acids, one amino acid more than *TaMOR-B* and *TaMOR-D*. The sequence similarities of *TaMOR-A*, *TaMOR-B*, and *TaMOR-D* amino acids and cDNAs were as high as 97.12% and 96.90%, respectively.

**Fig. 2.**
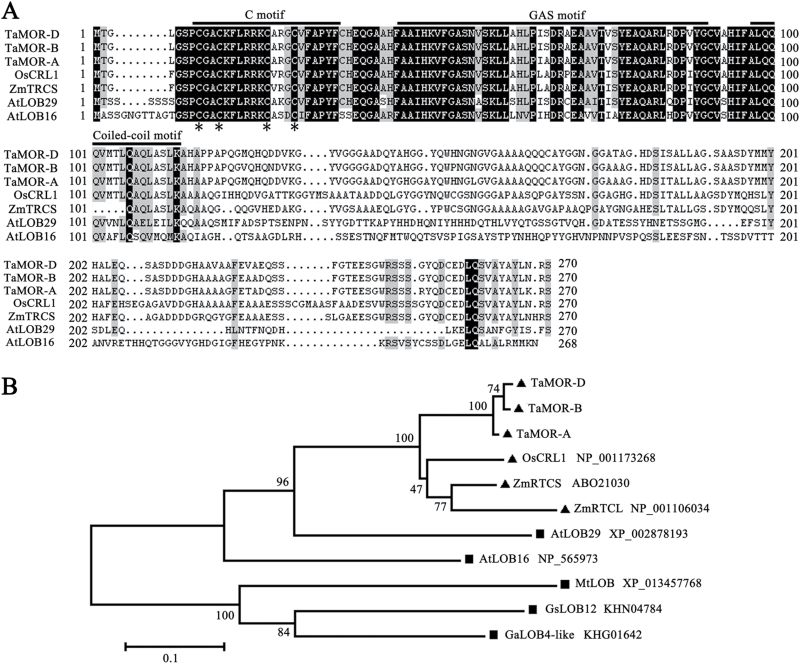
TaMOR belongs to the plant-specific LOB protein family. (**A**) Comparison of LBD proteins in different plant species. Common amino acid residues are shown in black background. The conserved C, GAS, and coiled-coil motifs are marked with overlines. Asterisks indicate the conserved Cys residues. (**B**) Phylogenetic tree of TaMORs and LOB proteins from other plant species. The tree was constructed by the neighbour-joining method with 1000 bootstrap replicates. Monocotyledonous species are indicated with filled triangles, dicotyledons are indicated with filled rectangles. Ta, *T. aestivum*; Os, *Oryza sativa*; Zm, *Zea mays*; At, *A. thaliana*; Mt, *Medicago truncatula*; Gs, *Glycine soja*; Ga, *Gossypium arboretum*.

TaMOR contains a plant-specific LOB domain, composed of a four-Cys motif, a GAS motif, and a coiled-coil motif. The LOB domain is highly conserved in LBD proteins from wheat, maize, rice, and Arabidopsis (Fig. 2A). A neighbour-joining phylogenetic tree was constructed to determine the relationship between TaMORs and their counterparts in other plant species (Fig. 2B). The TaMORs were classified in the same clade as LOB proteins from monocotyledons, including OsCRL1, ZmRTCS, and ZmRTCL.

Because the three *TaMORs* have extremely high similarities in amino acids, and almost identical LOB domains; the D genome member, *TaMOR-D*, was selected for further functional analysis.

### Genetic characteristic of *TaMORs* in wheat

**Fig. 3.**
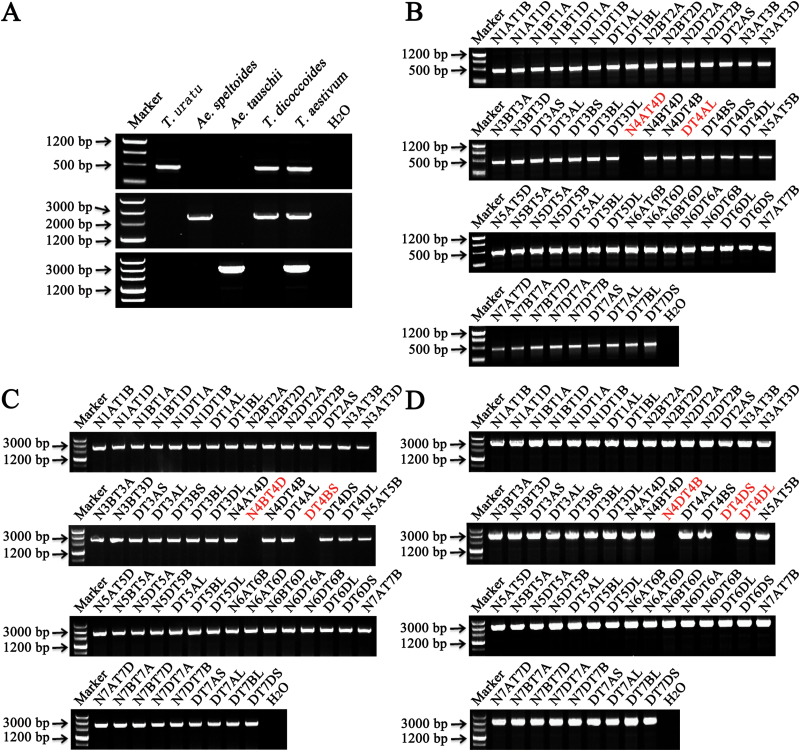
Chromosome locations of *TaMORs* genes in wheat. (**A**) Target fragments amplified by *TaMOR* allele-specific primer pairs (Supplementary Table S2) designed for the A, B, and D genomes. (**B, C, D**) *TaMOR-A*, *TaMOR-B*, and *TaMOR-D* were located on chromosomes 4AL, 4BL, and 4DL, respectively, using nulli-tetrasomic and ditelosomic lines of Chinese Spring. Marker, DNA marker Ⅲ (TransGen, Beijing). This figure is available in colour at *JXB* online, showing the target chromosomes in red font.

Allele-specific primers pairs (Fig. 3A, Supplementary Fig. S1, and Supplementary Table S2) were designed to probe the chromosome origin of different alleles. Furthermore, *TaMOR-A*, *TaMOR-B*, and *TaMOR-D* were located on chromosomes 4AL (Fig. 3B), 4BL (Fig. 3C), and 4DL (Fig. 3D) with sets of nulli-tetrasomic and ditelosomic lines of Chinese Spring.

### *TaMORs* are highly conserved in evolution and in wheat improvement

**Fig. 4.**
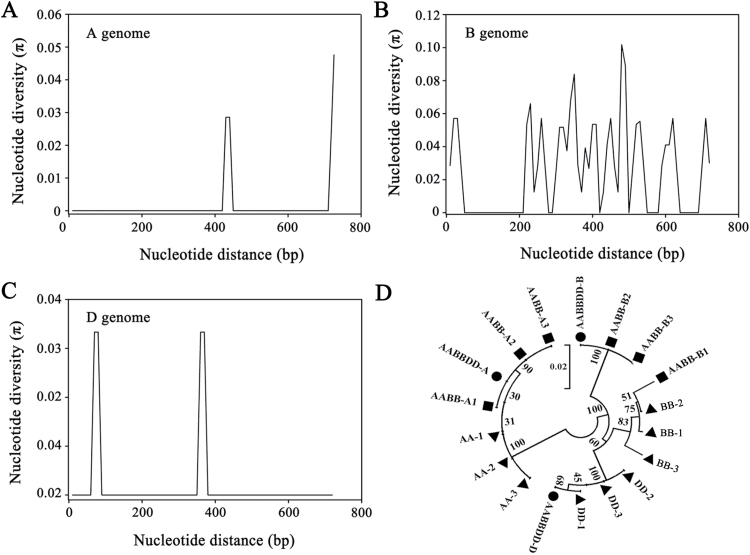
Homology of nucleotide sequences and nucleotide diversity. Thirteen accessions were used for sequence analysis (AA-1–AA-3, *T. urartu* 1–3; BB-1–BB-3, *Ae. speltoides* 1–3; DD-1–DD-3, *Ae. tauschii* 1–3; AABB-1–AABB-3, *T. dicoccoides* 1–3; AABBDD, *T. aestivum*. (**A, B, C**) The nucleotide acid diversity (π) of *TaMORs* in A, B, and D genomes in diploid, tetraploid, and common wheat accessions, respectively. (**D**) Phylogenetic tree of *TaMORs* in diploid, tetraploid, and common wheat accessions. The tree was constructed by the neighbour-joining method with 1000 bootstrap replicates.

To assess the influence of evolution, *TaMOR-A*, *TaMOR-B*, and *TaMOR-D* and their orthologs in diploid and tetraploid wild relative species were cloned and sequenced. *TaMORs* including coding and partial flanking regions were amplified from 34 modern hexaploid cultivars and 13 progenitor accessions using genome-specific primers. SNP analysis showed that there were six (Supplementary Table S3), 34 (Supplementary Table S4), and two (Supplementary Table S5) SNP sites in the *TaMORs* from the A, B, and D genomes, respectively (Fig. 4A–C). The sequencing materials were diploid, tetraploid, and hexaploid accessions. All sequences identified in common wheat were present in the corresponding genome donor species (Fig. 4D and Supplementary Fig. S2). Thus, *TaMORs* were highly conserved during evolution.

Scanning of 34 modern cultivars (Supplementary Table S1) using the allele-specific primers showed no nucleotide or amino acid differences in the A and D genomes. A single cultivar had two nucleotide polymorphisms (G/C and C/G) (Supplementary Fig. S3A) and one nucleotide difference (A/G) (Supplementary Fig. S3B) in the B genome, but the conserved domain was unchanged, indicating that both the nucleotide and amino acid sequences were highly conserved during wheat improvement.

### Subcellular localization and transcriptional activation activity of TaMOR-D protein

**Fig. 5.**
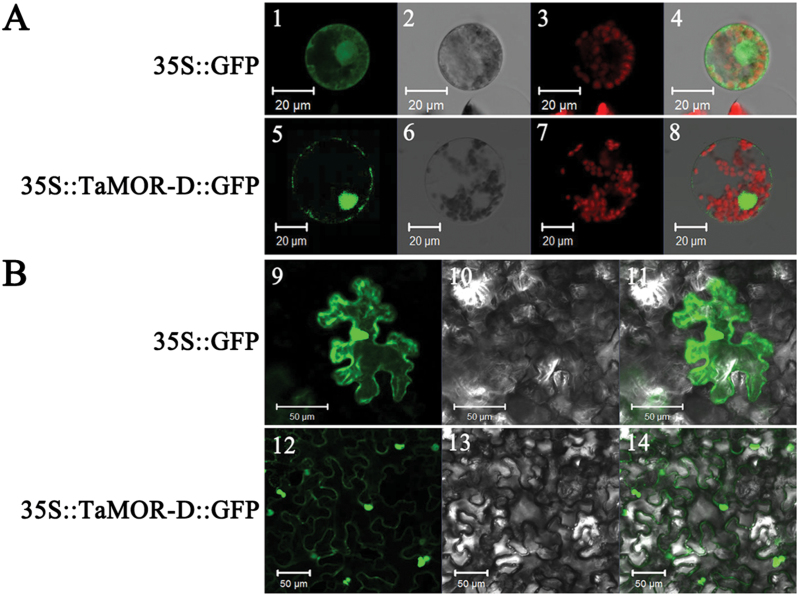
Subcellular localization of TaMOR-D in wheat protoplasts (**A**) and tobacco leaf cells (**B**). The vector control (35S::GFP) and fusion protein construct 35S::TaMOR-D::GFP were introduced into wheat protoplast and tobacco leaf cells, respectively. For wheat protoplast transformation, GFP was detected at 24h with a laser scanning confocal microscope; for tobacco, GFP was detected at 4 d. Images are in dark field (1, 5, 9, 12), bright field (2, 6, 10, 13), and combined (4, 8, 11, 14). Scale bars: 20 μm for wheat protoplasts, 50 μm for tobacco leaf cells. Chloroplasts are indicated by red auto-fluorescence (3, 7, 8) in the colour figure available at *JXB* online.

To determine the subcellular localization of TaMOR-D, the TaMOR-D-GFP fusion protein was expressed in wheat protoplasts and tobacco leaves. TaMOR-D was found in both the nucleus and cell membrane (Fig. 5).

Structure prediction suggested that *TaMOR-D* encodes a TF. To further test its role as a TF, transcriptional activation experiments were conducted using a modified yeast two-hybrid assay. As shown in Fig. 1B, transcriptional activation ability was detected only in fusion proteins containing P4 fragments. Deletion analysis indicated that the C-terminal of TaMOR-D was crucial for transcriptional activation activity.

### Expression pattern of *TaMORs* in wheat

**Fig. 6.**
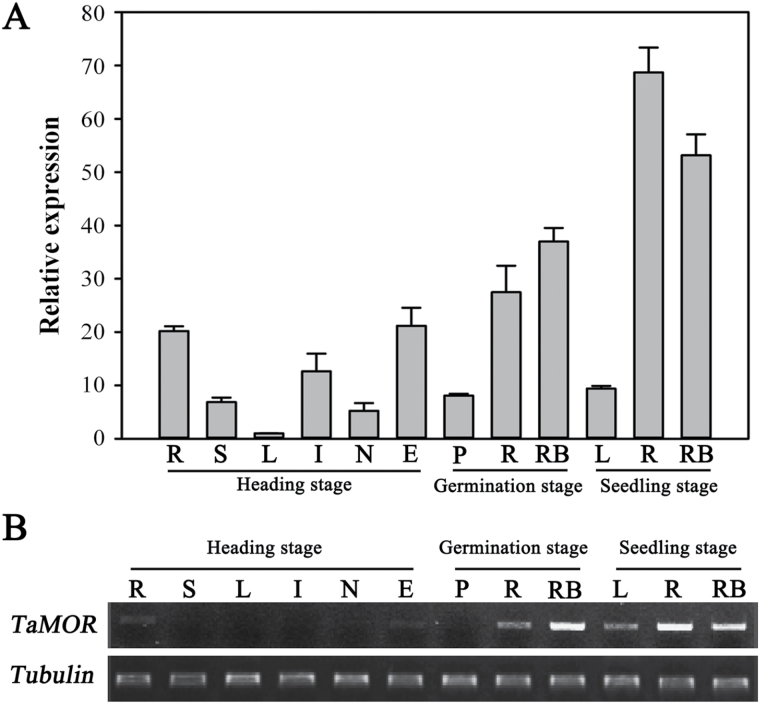
Expression patterns of *TaMORs* in wheat tissues at different developmental stages detected by qRT-PCR (**A**) and semi-quantitative RT-PCR (**B**). R, roots; S, leaf sheaths; L, leaf blades; I, internodes; N, nodes; E, ears; P, plumules; RB, root bases. Error bars represent the SD of triplicate reactions. The experiment was repeated three times with similar results.

Quantitative RT-PCR and semi-quantitative RT-PCR were performed to analyse the expression patterns of *TaMORs* in various organs and stages in wheat (Fig. 6). High expression levels were evident in roots and root initiation sites at all three stages of germination, seedling, and heading. High expression levels were also detected in ears at the heading stage. The expression of *TaMORs* corresponded well to areas and periods of tissue initiation, confirming that *TaMORs* are involved in initiation of wheat roots.

**Fig. 7.**
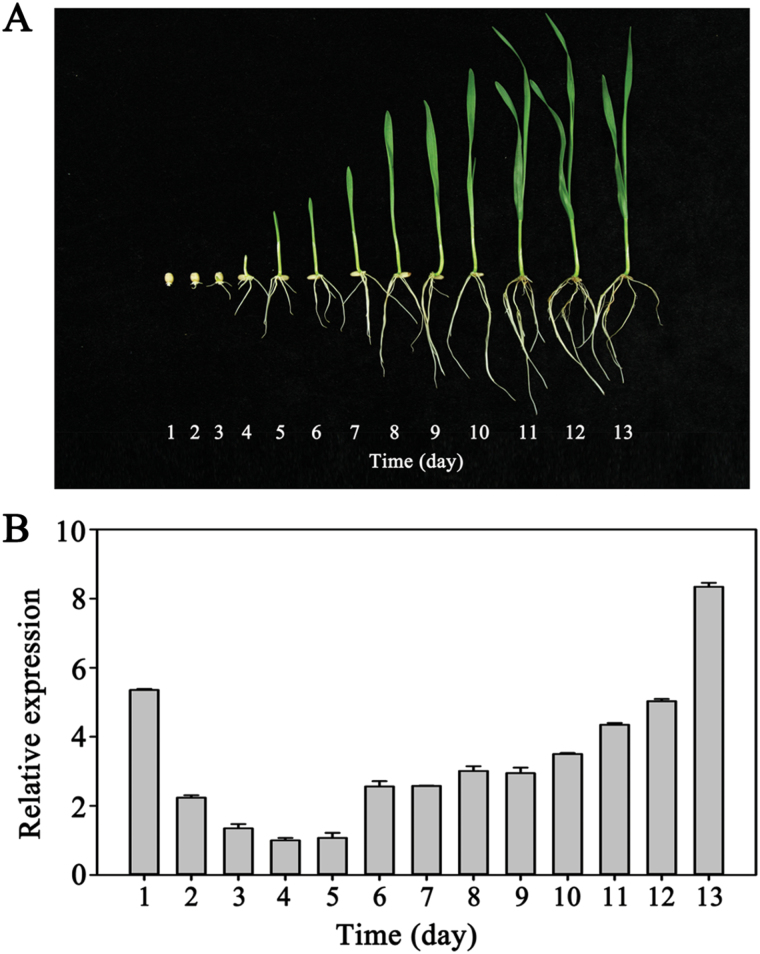
Expression pattern of *TaMORs* from germination to the seedling stage in wheat. (**A**) Wheat growth status from germination to seedling stage. Embryos and root bases were sampled for *TaMORs* expression analyses. (**B**) Expression patterns of *TaMORs* in embryos and root bases after seed germination. *Tubulin* activity was used as an internal control. Vertical columns indicate relative transcript levels. Error bars represent the SD of triplicate reactions. The experiment was repeated three times with similar results. This figure is available in colour at *JXB* online.

The expression patterns of *TaMORs* from germination to the seedling stage were identified by qRT-PCR. Primary root system initiation was detected from the first day of germination to the seedling stage when secondary roots began to develop (Fig. 7A). A U-shape expression curve was identified in embryos and root bases over a 13-day period. Expression peaked on the first day at primary root initiation sites and on the 13th day at secondary root initiation sites (Fig. 7B).

### Auxin signalling regulates *TaMOR* expression in wheat

**Fig. 8.**
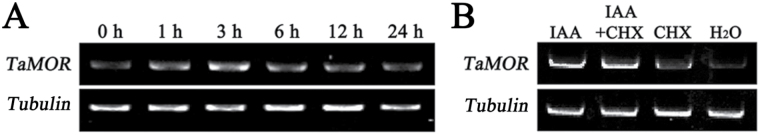
*TaMORs* activity is induced in wheat by auxin. (**A**) Seedlings were treated with 1 μM IAA, and whole plants were sampled at various time points; time 0h represents the time immediately prior to treatment. *Tubulin* was used as an internal control. (**B**) Effects of auxin and CHX on *TaMORs* transcription. Seedlings were treated with or without 1 μM IAA and 50 μM CHX.

The response of *TaMORs* to auxin was detected by semi-quantitative RT-PCR. *TaMORs* were induced within 1h and peaked 3h after treatment with IAA, and then gradually decreased (Fig. 8A). The effect of the protein synthesis inhibitor CHX on auxin-dependent induction of *TaMORs* was also examined. Auxin-dependent induction of *TaMORs* was not inhibited by CHX (Fig. 8B), suggesting that *TaMORs* have a role in auxin signalling and that *de novo* protein synthesis might not be required for auxin-induced gene expression.

### Identification of TaMOR-interacting proteins

To decipher the mechanism of TaMORs in regulating root initiation, the N-terminal of TaMORs (1-108AA), which lacks transcriptional activation activity, was used as bait to screen a yeast two-hybrid cDNA library of wheat to identify TaMOR-interacting proteins. Yeast two-hybrid screening yielded two candidate clones. One of them, a pentatricopeptide repeat (PPR) protein designated TaMRRP, was verified as the interacting protein.

**Fig. 9.**
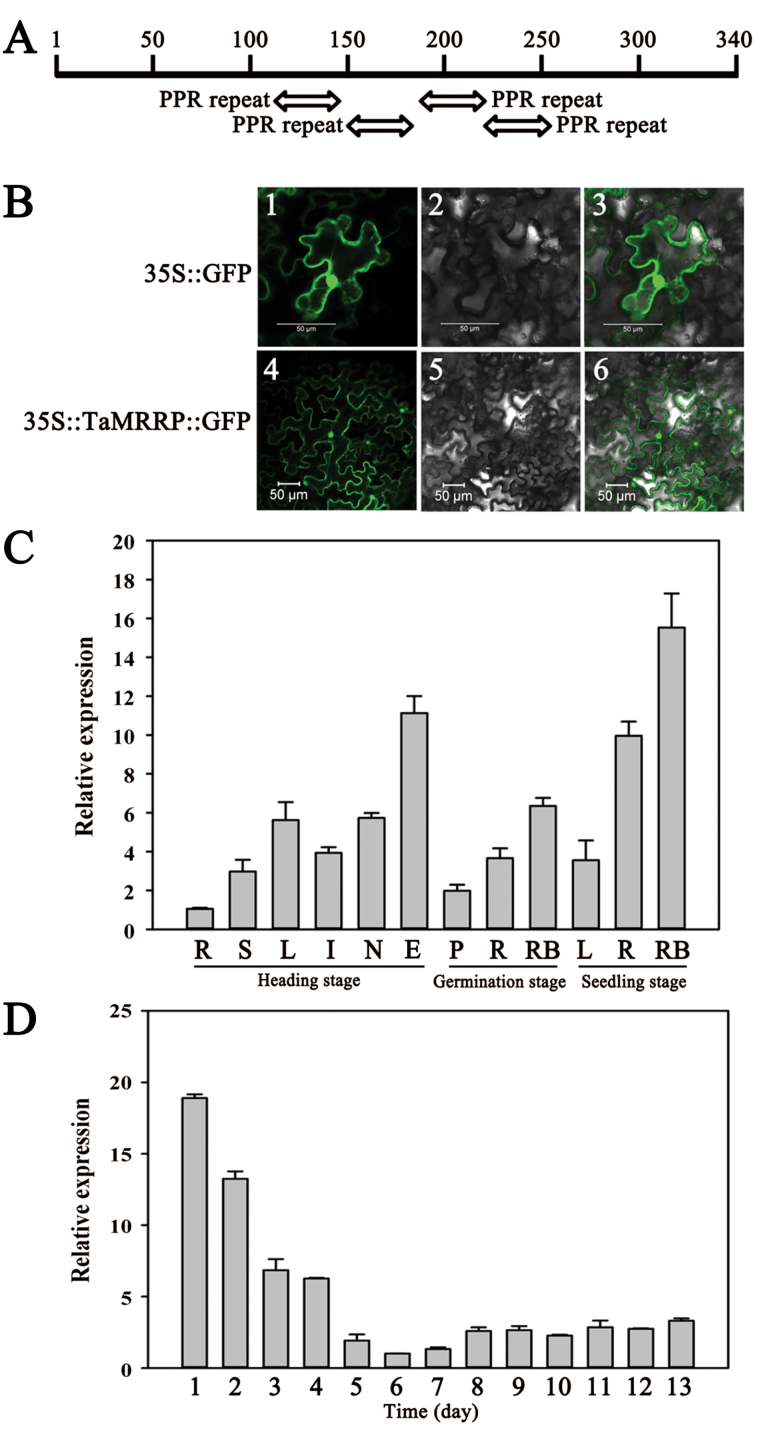
Characteristics of *TaMRRP* in wheat. (**A**) Structure of TaMRRP. Pentatricopeptide repeat domains (PPR motifs) are indicated according to the amino acid sequence. (**B**) Subcellular localization of TaMRRP in tobacco cells. The vector control (35S::GFP) and fusion proteins (35S::TaMRRP::GFP) were introduced into tobacco leaf cells and fluorescence was observed with a laser scanning confocal microscope. Images are in dark field (1, 4), bright field (2, 5), and combined (3, 6). Scale bar, 50 μm. (**C**) Expression patterns of *TaMRRP* in different wheat tissues at different developmental stages. R, roots; S, leaf sheaths; L, leaf blades; I, internodes; N, nodes; E, ears; P, plumules; RB, root bases. (**D**) Expression patterns of *TaMRRP* during germination and seedling development after seed germination. *Tubulin* was used as the internal control. Error bars represent the SD of triplicate reactions. The experiment was repeated three times with similar results. This figure is available in colour at *JXB* online.

TaMRRP, a protein of 340 amino acids with a predicted pI of 5.8 and molecular weight of 38.76kDa, possesses a four-tandem PPR motif (Fig. 9A). A *TaMRRP-GFP* construct was transferred to tobacco leaf cells, and GFP was detected in both the nucleus and cell membranes (Fig. 9B). Subcellular localization of TaMRRP was consistent with that of TaMOR-D, suggesting that *TaMRRP* might interact with *TaMOR-D in vivo*.

High expression of *TaMRRP* was also evident in roots and root bases at the germination and seedling stages, and low expression occurred in roots at the heading stage (Fig. 9C). This result was almost consistent with the presence of *TaMORs*. However, *TaMRRP* was also highly expressed in ears at the heading stage, suggesting that *TaMRRP* might also be involved in ear development. The expression of *TaMRRP* decreased gradually from germination to the seedling stage (from the first to 13th days) (Fig. 9D), which was quite similar to the *TaMOR* expression pattern at the early stage (from the first to fifth days).

### TaMOR-D physically interacts with TaMRRP

**Fig. 10.**
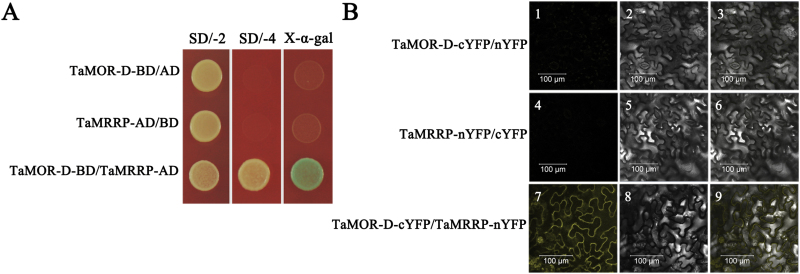
TaMOR-D interacts with TaMRRP. (**A**) TaMOR-D interacts with TaMRRP in yeast. The transformants were placed on SD/−Trp/−Leu (SD/−2) medium to examine growth. Protein–protein interactions were assessed on SD/−Ade/−His/−Trp/−Leu (SD/−4) medium and further confirmed by monitoring α-galactosidase activity. (**B**) TaMOR-D interacts with TaMRRP in tobacco. Agrobacteria carrying the indicated construct pairs were injected into tobacco leaves. Fluorescence was detected 3 d after transformation. Images are in dark field (1, 4, 7), bright field (2, 5, 8), and combined (3, 6, 9). Scale bar, 100 μm. This figure is available in colour at *JXB* online.

A yeast two-hybrid assay indicated that TaMRRP interacted with TaMOR-D (Fig. 10A). A BiFC assay was performed to test whether the two proteins interacted in plants. A yellow florescence signal was detected only when TaMRRP-nYFP and TaMOR-D-cYFP were co-expressed in tobacco leaf cells. YFP was present on the cell membrane (Fig. 10B), consistent with a subcellular localization of both proteins. We concluded that TaMOR-D probably interacts with TaMRRP on the cell membrane.

### *TaMOR-D* overexpression causes increased lateral root development in Arabidopsis

**Fig. 11.**
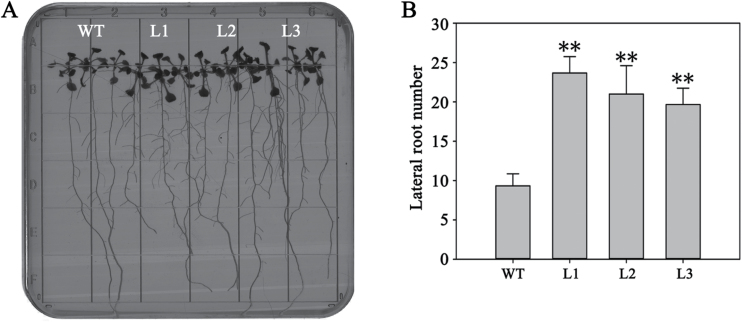
Overexpression of *TaMOR-D* leads to more lateral roots in Arabidopsis. (**A**) Lateral root phenotypes of *TaMOR-D*-overexpressing Arabidopsis lines. (**B**) Comparison of lateral root number between WT and transgenic lines. WT, wild type; L1, Line 1; L2, Line 2; L3, Line 3. The error bars denote 1 SD; n = 9. **P < 0.01.

*TaMOR-D* was transferred into Arabidopsis to assess the function of *TaMOR* in a dicot. Three T_3_ transgenic lines of *TaMOR-D* with similar transcription levels were used for phenotyping (Supplementary Fig. S4A). The *TaMOR-D* overexpressing lines had significantly more lateral roots than WT (Fig. 11).

### Phenotypes of *TaMOR-D* transgenic rice lines

**Fig. 12.**
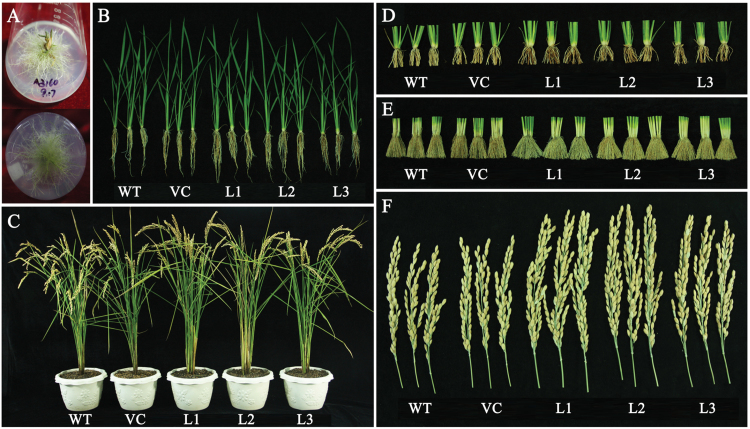
Phenotypes of *TaMOR-D*-overexpressing rice plants at different developmental stages. Some of the T_0_ generation *TaMOR-D*-overexpressing plants had abundant crown roots and did not grow normally (**A**). Phenotypes of transgenic lines and WT plants at the seedling (**B**) and adult (**C**) growth stages. Transgenic lines had more base roots at the seedling (**D**) and adult growth stages (**E**). The main panicle size of transgenic plants was much larger than that of the controls (**F**). WT and VC are the controls; VC indicates plants transformed with the empty pCUbi1390 vector. This figure is available in colour at *JXB* online.

**Fig. 13.**
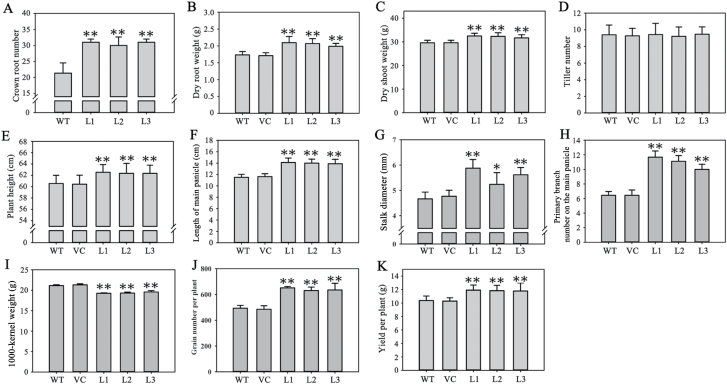
Comparison of biological and agronomic traits between *TaMOR-D*-overexpressing lines and control rice plants. Traits were measured at the seedling stage (A) and adult (B–K) stages. Crown root number (**A**), dry root weight (**B**), dry shoot weight (**C**), tiller number (**D**), plant height (**E**), length of main panicle (**F**), stalk diameter (**G**), primary branch number on the main panicle (**H**), 1000-kernel weight (**I**), grain number per plant (**J**), and yield per plant (**K**). The error bars denote 1 SD; n = 10. *P < 0.05; **P < 0.01.

Twenty-one transgenic lines (T_0_ generation) were obtained; however, 16 lines with excessive crown roots and extreme dwarfism failed to produce seeds, probably because of ultra-high expression of *TaMOR-D* (Fig. 12A). The remaining lines grew normally, and did not show a significant change in expression level of the target gene (Supplementary Fig. S4B). These transgenic lines had significantly more roots and larger stalk diameters than WT and the empty vector control at both the seedling and mature growth stages (Figs 12B–E, 13A). Furthermore, the main panicles of the transgenic rice lines were significantly longer, and the grain numbers per panicle were also significantly higher than those in the controls (Fig. 12F).

Several biological and agronomic traits were measured to characterize the phenotypes of the transgenic plants, including dry root weight, dry shoot weight, tiller number, plant height, length of main panicle, stalk diameter, primary branch number on the main panicle, 1000-kernel weight, grain number per plant, and grain yield per plant (Fig. 13B–K). All measured traits except tiller number and 1000-kernel weight were significantly higher than those of the controls. The transgenic rice lines had larger root systems, that is, more crown roots at seedling stage and heavier dry root weight at adult stage. The transgenic rice lines also had better yield-related traits than the WT plants, such as longer main panicle, more primary branches on the main panicle, a higher grain number per plant, and a higher yield per plant. Further comparisons indicated that the increased plant height was due to larger panicles with more primary branches. Although the 1000-kernel weights of the transgenic lines were lower than the controls, the grain yield per plant was still significantly higher than the controls.

## Discussion

Optimizing the root architecture of crop plants is regarded as an important objective for a new green revolution because of its crucial roles in anchorage, soil resource acquisition, and the establishment of plant-microbial communities (Villordon *et al.*, 2014). Therefore it is essential to understand the molecular mechanisms of root growth and development for future crop improvement. Some genes involved in root development have been cloned in Arabidopsis, rice, and maize (Overvoorde *et al.*, 2010); however, studies so far in wheat have mainly focused on the molecular mapping of root trait QTLs (Cao *et al.*, 2014; Christopher *et al.*, 2013). Here, we have identified that *TaMOR* is involved in root initiation in wheat, and found that its overexpression not only increased root number but also increased grain yield. Our studies therefore offer opportunities for crop improvement by regulating root architecture.

### TaMORs are highly conserved in evolution and crop improvement

TaMORs belong to the plant-specific LOB protein family and contain a typical LOB domain. The LOB domains among TaMORs and their orthologues are highly conserved in structure. Many wheat genes are represented by series of haplotypes that develop during evolution and domestication. For example, wheat spikelet development gene *TaMOC1* formed two haplotypes during wheat domestication and breeding in China (Zhang *et al.*, 2015). Wheat sucrose synthase genes *TaSus1* and *TaSus2* formed various haplotypes during a century of wheat breeding (Hou *et al.*, 2014). However, *TaMOR* sequences across the A, B, and D genomes have been highly conserved during evolution and domestication. The high degree of conservation in modern cultivars implies that *TaMORs* play essential roles in growth and development, and that any variation in the conserved domain has adverse effects on plant growth.

### TaMOR functions as a transcription factor

LBD proteins belong to a plant-specific TF family. Arabidopsis LBD/ASL TFs, for example, are involved in the regulation of callus formation (Fan *et al.*, 2012). The TFs RTCS and RTCL can bind to *LBD*-downstream promoters (Taramino *et al.*, 2007; Xu *et al.*, 2015). LBD18, functioning as a specific DNA-binding transcriptional activator, regulates the expression of *EXP14* by directly binding to its promoter (Lee *et al.*, 2013). Our experimental data show that TaMOR-D is localized in the cell nucleus and that its C-terminal possesses transcriptional activation activity.

### *TaMORs* are induced by auxin and are involved in root initiation

Previous studies of root in mutants showed that plant-specific *LBD* genes are induced by auxin and are involved in root initiation (Inukai *et al.*, 2005; Taramino *et al.*, 2007; Feng *et al.*, 2012a). *RTCS* and *RTCL* genes display highly correlated spatiotemporal expression patterns in maize roots (Xu *et al.*, 2015). *TaMOR* expression patterns during germination and seedling development and in different tissues are consistent with the time of root initiation and occur at root initiation sites. Our results indicate that *TaMORs* play similar roles in root initiation to the earlier reported *OsCRL1* and *ZmRTCS* genes (Inukai *et al.*, 2005; Taramino *et al.*, 2007).

AuxREs, which contain the TGTCTC motif, are the binding sites of ARFs. AuxREs have been identified in promoters of early auxin responsive genes, and ARFs bind to AuxREs to regulate transcription of these genes (Hagen and Guilfoyle, 2002). Four putative AuxREs were identified in a 2kb promoter region of *TaMOR-D* (Supplementary Fig. S5). The presence of AuxREs in the *TaMOR-D* promoter confirms that *TaMORs* can be induced by auxin in a similar way to the early auxin-inducible gene in Arabidopsis (Abel *et al.*, 1995), suggesting *de novo* protein synthesis is not required for *TaMOR* induction by auxin.

### TaMOR interacts with TaMRRP on the cell membrane

PPR proteins belong to one of the largest and most complex gene families in plants and have been widely implicated in many crucial functions, including organelle biogenesis and development (Saha *et al.*, 2007). PPR protein features a degenerate 35-amino-acid repeat motif, often arranged in tandem arrays of 2–27 repeats per peptide (Lurin *et al.*, 2004; Raczynska and Augustyniak, 2005). Some PPR proteins play roles as adaptors and partner in protein–protein interactions. PPR proteins are usually located in organelles, except for *GLUTAMINE-RICH PROTEIN 23* (*GRP 23*), which targets the nucleus (Fisk *et al.*, 1999; Ding *et al.*, 2006). The specific expression pattern of the *LATERAL ORGAN JUNCTION* (*LOJ*) gene encoding a protein with 19 PPR motifs suggests it is involved in lateral organ development and boundary formation (Prasad *et al.*, 2005). In our research, the similar expression profiles of *TaMORs* and *TaMRRP* identified in roots and root bases, as well as their expression patterns at germination to the early seedling stage (first to fifth day), suggest that *TaMORs* and *TaMRRP* are jointly involved in root initiation and development. Moreover, higher expression of *TaMRRP* in ears implies involvement in ear development.

Our data indicate that both TaMOR-D and TaMRRP are localized in the nucleus and on the cell membrane, and that TaMRRP is a target of TaMOR-D. Previous research showed that the tandem PPR motifs might form a superhelix enclosing a groove or tunnel, and that the superhelix structure acts as a protein-binding motif and can bind to specific ligands (Small and Peeters, 2000). The fact that TaMRRP contains four typical PPR motifs leads us to speculate that TaMRRP might interact with TaMOR-D by binding to the C-terminal leucine-zipper-like region. The exact manner of interaction needs to be further investigated.

### *TaMOR* is a promising candidate gene for root architecture improvement and grain yield enhancement

Previous studies showed that more directed selection for specific root architecture traits could enhance yields (Wasson *et al.*, 2012). The cloning and characterization of *DRO1* shed new light on simultaneous root system improvement and grain yield enhancement in crops (Uga *et al.*, 2013; Villordon *et al.*, 2014). Modelling studies in Australia showed that selection for deeper, more effective roots significantly improved the capture of water and nitrogen (Manschadi *et al.*, 2006; Lilley and Kirkegaard, 2011). Changing the root branching distribution from linear to strongly exponential improved P uptake by 142% for low-P soils when root mass was kept constant between simulations (Heppell *et al.*, 2015). Our research indicates that overexpression of *TaMOR-D* leads to improved root architecture in rice and increased lateral roots in Arabidopsis, indicating that *TaMOR-D* could be used in both dicots and monocots to improve root architecture. An improved root system implies an increased root number and root surface area, which should enhance the capacity of water absorption and nutrient acquisition, as well as improve plant anchorage in shallow soils. The transgenic rice lines had a distinct advantage on the yield-related traits, including longer main panicle, more primary branches on the main panicle, more grains per plant, and higher grain yield per plant. So, transgenic rice plants were significantly superior to control plants in biomass, stalk diameter, panicle size, and grain yield, suggesting that *TaMOR-D* has potential to improve grain yield. Although the transgenic rice had a larger panicle and higher total kernel weight, the plants remained erect at the mature stage owing to the thicker culms and therefore likely possessed improved lodging resistance under field conditions. However, we also observed lethal effects of *TaMOR-D* overexpression in some transformants. A similar phenomenon was observed with other auxin-responsive genes in petunia (*Petunia hybrida*) and rice (Tobena-Santamaria *et al.*, 2002; Yamamoto *et al.*, 2007). In our study, the CaMV 35S promoter was used to promote the expression of *TaMOR-D* in rice. This promoter is a constitutive, high-expression promoter. To avoid the lethal effect, a more appropriate promoter and favourable expression level might be considered for future use. Based on the present results, we predict that overexpressing *TaMOR-D* lines could contribute to a breeding strategy for improved root architecture and hence increased crop production.

## Supplementary data

Supplementary data are available at *JXB* online.

Figure S1. The cloning regions of *TaMORs* using allele-specific primers.

Figure S2. Homology of genomic nucleotide sequences.

Figure S3. Sequence alignment of *TaMOR-B* in four modern wheat cultivars.

Figure S4. Relative expression levels of *TaMOR-D* in transgenic *Arabidopsis* (A) and rice lines (B).

Figure S5. Promoter structure of *TaMOR-D*.

Table S1. Cultivars used for the target gene sequence analysis.

Table S2. Primers used in the research.

Table S3. The SNP sites of *TaMOR-A* in A genome.

Table S4. The SNP sites of *TaMOR-B* in B genome.

Table S5. The SNP sites of *TaMOR-D* in D genome.

Supplementary Data
