# Supplementary material for: Overexpression of wheat gene TaMOR improves root system architecture and grain yield in Oryza sativa
Source: J Exp Bot. 2016 May 26;67(14):4155–67. doi: 10.1093/jxb/erw193 (PMC5301925; doi:10.1093/jxb/erw193)
Supplement: Supplementary Data [file supp_67_14_4155__index.html]

Overexpression of wheat gene TaMOR improves root system architecture and grain yield in Oryza sativa — Overexpression of wheat gene TaMOR improves root system architecture and grain yield in Oryza sativa — Supplementary Data 

# Overexpression of wheat gene *TaMOR* improves root system architecture and grain yield in *Oryza sativa*

## Supplementary Data

Data files

- Supplementary\_Figure\_S1\_S5\_\_Table\_S1\_S5.pdf - Supplementary Data
