# Supplementary material for: Overexpression of wheat gene TaMOR improves root system architecture and grain yield in Oryza sativa
Source: J Exp Bot. 2016 May 26;67(14):4155–67. doi: 10.1093/jxb/erw193 (PMC5301925; doi:10.1093/jxb/erw193)
Supplement: Supplementary Data [file supp_erw193_Supplementary_Figure_S1_S5__Table_S1_S5.pdf]

Overexpression of wheat gene *TaMOR* improves root system architecture and grain yield in rice

Bo Li, Dan Liu, Qiaoru Li, Xinguo Mao, Ang Li, Jingyi Wang, Xiaoping Chang, Ruilian Jing

National Key Facility for Crop Gene Resources and Genetic Improvement/Institute of Crop Science, Chinese Academy of Agricultural Sciences, Beijing 100081, China

Author for correspondence:
Ruilian Jing
Tel: +86 1082105829
Fax: +86 1082105829
Email: jingruilian@caas.cn

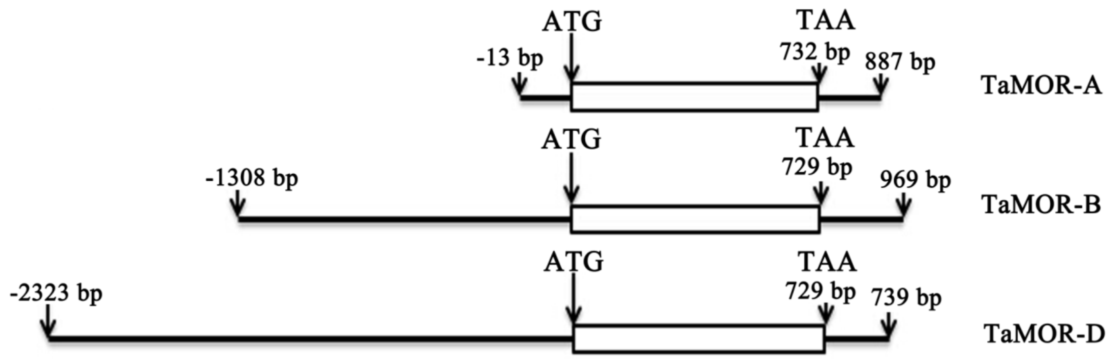

Figure S1. The cloning regions of *TaMORs* using allele-specific primers.

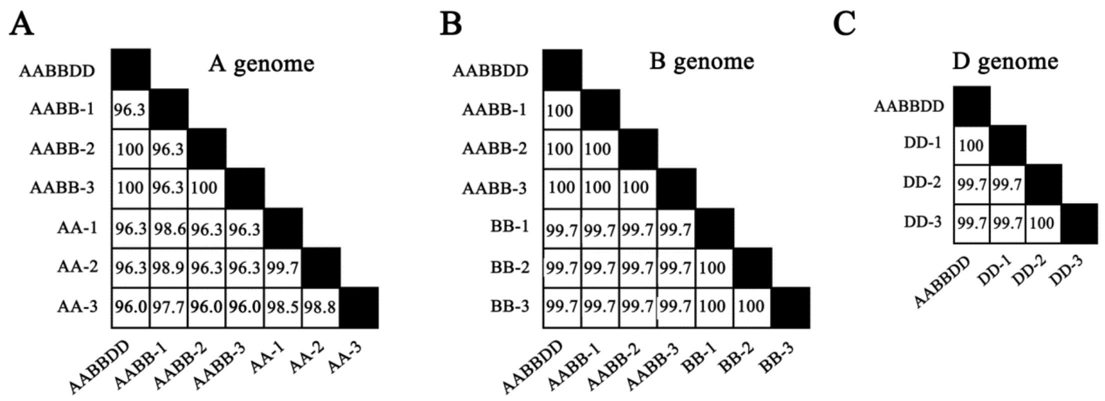

Figure S2. Homology of genomic nucleotide sequences. (A, B, C) Homology of A, B and D genomic nucleotide sequences between diploid accessions (AA, BB, DD), tetraploid accessions (AABB) and common wheat (AABBDD), respectively.

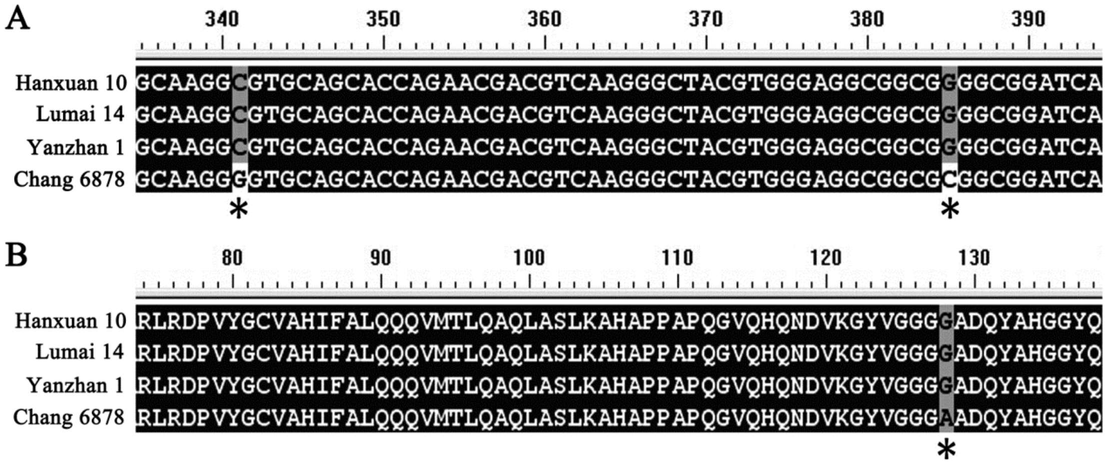

Figure S3. Sequence alignment of *TaMOR-B* in four modern wheat cultivars. (A) Alignment of genomic sequences among 34 unrelated modern cultivars (Table S1). (B) Alignment of amino acid sequences among 34 unrelated modern cultivars (Table S1). Hanxuan 10, Lumai 14, Yanzhan 1 and Chang 6878 are shown as examples. Shared nucleotide and amino acid sequences are shown in black background. Asterisks represent the polymorphic sites.

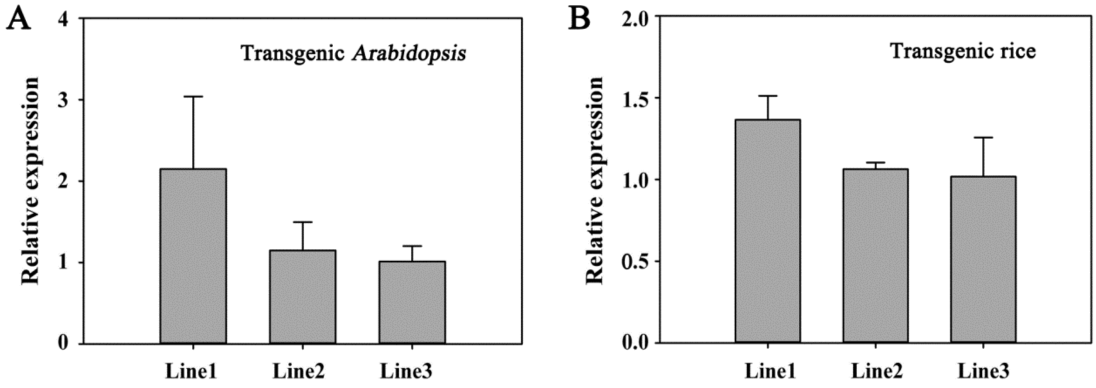

Figure S4. Relative expression levels of *TaMOR-D* in transgenic *Arabidopsis* (A) and rice lines (B). The error bars represent the SD of triplicate reactions.

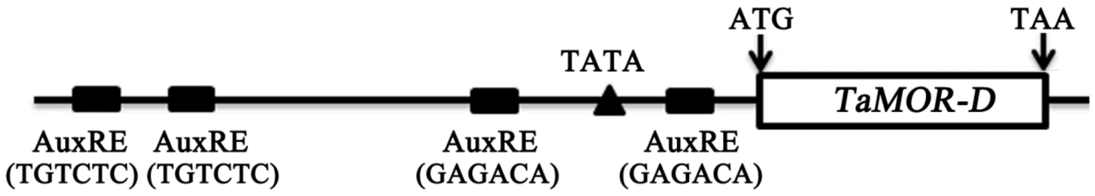

Figure S5. Promoter structure of *TaMOR-D*. There are four putative auxin response elements (AuxRE) and one TATA box in the 2 kb *TaMOR-D* promoter region.

Table S1. Cultivars used for the target gene sequence analysis

No.	Cultivar	Origin	Time of release
1	Beijing 10	Beijing, China	1960s
2	Hanxuan 10	Shanxi, China	1960s
3	An 85 Zhong 124-1	Beijing, China	1970s
4	Dan R 8093	Beijing, China	1970s
5	Jingpin 10	Beijing, China	1970s
6	Jimai 6	Hebei, China	1970s
7	Changle 5	Shandong, China	1970s
8	Dali 1	Shaanxi, China	1970s
9	Beijing 14	Beijing, China	1980s
10	Fengkang 13	Beijing, China	1980s
11	Jinghe 8922	Beijing, China	1980s
12	Jin 2148-7	Fujian, China	1980s
13	Changwu 131	Shaanxi, China	1980s
14	04-030	Beijing, China	1990s
15	04-044	Beijing, China	1990s
16	Beijing 8686	Beijing, China	1990s
17	Jing 411	Beijing, China	1990s
18	Jimai 41	Hebei, China	1990s
19	Neixiang 188	Henan, China	1990s
20	Yanzhan 1	Henan, China	1990s
21	Pandas	Italy	1990s
22	Lumai 14	Shandong, China	1990s
23	Chun 9th-25	CIMMITY, Mexico	2000s
24	Chun 9th-5-1	CIMMITY, Mexico	2000s
25	Chun 9th-50-1	CIMMITY, Mexico	2000s
26	Chang 6878	Shanxi, China	2000s
27	Liangxing 99	Shandong, China	2000s

28	Linkang 5108	Shanxi, China	2000s
29	Baiqimai	Gansu, China	Landrace
30	Baicaomai	Henan, China	Landrace
31	Ziganbaimangxian	Henan, China	Landrace
32	Cangzhouxiaomai	Hebei, China	Landrace
33	Hongheshang	Shanxi, China	Landrace
34	Chinese Spring	Sichuan, China	Landrace

Table S2. Primers used in the research

Primer set	Primer sequence (5′-3′)		Amplified target
P-TaMOR	Forward	TCGGTTGATGAGCATGACG	DNA and cDNA of
	Reverse	GCTGGCTAGGACTAGAACCTGA	D, B and A genome
P-TaMOR-D	Forward	GCTAATGTGCTGCATACGGT	D genome-specific
	Reverse	GTAGTGGTTCTTACGAGCGATT	
P-TaMOR-B	Forward	CTCGTGGGAATGTCTGATGC	B genome-specific
	Reverse	CTGTCTAGTCAACTAGCTAGGTTCC	
P-TaMOR-A1	Forward	TCGGTTGATGAGCATGACG	A genome-specific
	Reverse	GAGACCGTTGTGCCACTG	
P-TaMOR-A2	Forward	CCAGTGGCACAACGGTCTC	A genome-specific
	Reverse	TGATCGATCGATCGACGCA	
P-TaMRRP	Forward	GGAGTTCTGGGAGCTGATCC	cDNA of *TaMRRP*
	Reverse	CACCATAATCTCACCAACAATTT	
P-TaMRRP-SD	Forward	TACCGCGTCGTGAGGAAGTT	qRT-PCR
	Reverse	CTCAGCCTCCATTTCATCGA	
P-TaMOR-D-At	Forward	TCCCCCGGGATGACGGGACTTGGGTC	Expression vector
	Reverse	CGGGATCCTTACGAGCGATTTAGGTACG	construction
P-TaMOR-D-Os	Forward	CGGGATCCATGACGGGACTTGGGTCG	Expression vector
	Reverse	GGACTAGTTTACGAGCGATTTAGGTACGC	construction
P-TaMOR-RT	Forward	GTCTTTGCGCCCTACTTCTG	qRT-PCR and
	Reverse	TCATGACCTGCTGCTGGAG	RT-PCR
P-Tubulin	Forward	TGAGGACTGGTGCTTACCGC	qRT-PCR and
	Reverse	GCACCATCAAACCTCAGGGA	RT-PCR in wheat
P-TaMOR-D-1300	Forward	CCCAAGCTTATGACGGGACTTGGGTCG	Expression vector
	Reverse	TCCCCCGGGCGAGCGATTTAGGTACGCAT	construction
P-TaMRRP-1300	Forward	CCCAAGCTTATGAAGGCCGGCGAGC	Expression vector
	Reverse	CGGGGTACCAGCAGTAGCCTGAACTTTTTTTG	construction
P-TaMOR-D-BD	Forward	CGGAATTCATGACGGGACTTGGGTCG	Expression vector

	Reverse	CGGGATCCTTAGGCGTGCGCCTTGAGC	construction
P-TaMRRP-AD	Forward	CGGAATTCATGAAGGCCGGCGAGC	Expression vector
	Reverse	CGGGATCCTTAAGCAGTAGCCTGAACTTTTTTTG	construction
P-TaMOR-D-cYFP	Forward	GGACTAGTATGACGGGACTTGGGTCG	Expression vector
	Reverse	GGGGTACCCGAGCGATTTAGGTACGCAT	construction
P-TaMRRP-nYFP	Forward	GGACTAGTATGAAGACCGGCGAGC	Expression vector
	Reverse	GGGGTACCAGCAGTAGCCTGAACTTTTTTTG	construction

Table S3. The SNP sites of *TaMOR-A* in A genome

No.	Site (bp)	AABBDD	AABB-1	AABB-2	AABB-3	AA-1	AA-2	AA-3
1	434	T	A	T	T	A	A	A
2	469	G	G	G	G	T	G	G
3	579	C	C	C	C	C	C	T
4	583	G	G	G	G	G	G	C
5	636	A	A	A	A	A	G	A
6	723	G	T	G	G	G	T	T

Seven accessions were used for sequence analysis (AABBDD, *T. aestivum*; AABB-1 ~ AABB-3, *T. dicoccoides* 1 ~ 3; AA-1 ~ AA-3, *T. urartu* 1 ~ 3).

Table S4. The SNP sites of *TaMOR-B* in B genome

No.	Site (bp)	AABBDD	AABB-1	AABB-2	AABB-3	BB-1	BB-2	BB-3
1	12	G	T	G	G	T	T	T
2	27	T	C	T	T	C	C	C
3	39	T	C	T	T	C	C	C
4	225	C	C	C	C	G	G	G
5	228	C	C	C	C	G	G	G
6	252	G	C	G	G	C	C	C
7	267	A	G	A	A	G	G	G
8	319	G	C	G	G	G	C	C
9	321	C	C	C	C	C	C	T
10	329	C	T	C	C	C	C	C
11	332	C	C	C	C	C	C	T
12	342	C	G	C	C	G	G	G
13	343	G	A	G	G	A	A	A
14	355	A	G	A	A	G	G	G
15	378	A	G	A	A	A	A	A
16	386	G	C	G	G	C	C	C
17	401	C	C	C	C	G	G	G
18	410	G	G	G	G	C	C	C
19	437	T	C	T	T	T	T	T
20	446	C	T	C	C	T	T	T
21	453	A	G	A	A	G	G	G
22	475	G	G	G	G	G	G	A
23	483	C	T	C	C	T	T	T
24	489	A	C	A	A	C	C	C
25	490	G	A	G	G	A	A	T
26	516	G	A	G	G	A	A	G
27	528	G	T	G	G	T	T	G
28	537	A	C	A	A	C	C	C
29	591	G	A	G	G	A	A	A
30	603	G	G	G	G	G	G	C
31	618	A	G	A	A	G	G	G
32	624	C	G	C	C	G	G	G
33	705	C	G	C	C	G	G	G
34	717	T	A	T	T	A	A	A

Seven accessions were used for sequence analysis (AABBDD, *T. aestivum*; AABB-1 ~ AABB-3, *T. dicoccoides* 1 ~ 3; BB-1 ~ BB-3, *Ae. speltoides* 1 ~ 3).

Table S5. The SNP sites of *TaMOR-D* in D genome

No.	Site (bp)	AABBDD	DD-1	DD-2	DD-3
1	72	T	T	C	C
2	366	A	A	G	G

Four accessions were used for sequence analysis (AABBDD, *T. aestivum*; DD-1 ~ DD-3, *Ae. tauschii* 1 ~ 3).
